# Nutritional and medical food therapies for diabetic retinopathy

**DOI:** 10.1186/s40662-020-00199-y

**Published:** 2020-06-18

**Authors:** Ce Shi, Peng Wang, Shriya Airen, Craig Brown, Zhiping Liu, Justin H. Townsend, Jianhua Wang, Hong Jiang

**Affiliations:** 1 Department of Ophthalmology, Bascom Palmer Eye Institute, University of Miami Miller School of Medicine, 1638 NW 10th Avenue, McKnight Building - Room 202A, Miami, FL 33136 USA; 2 School of Ophthalmology and Optometry, Wenzhou Medical University, Wenzhou, Zhejiang, China; 3 Department of Ophthalmology, Shanghai General Hospital, Shanghai Jiaotong University School of Medicine, Shanghai, China; 4 College of Arts and Sciences, University of Miami, Miami, FL USA; 5 Department of Ophthalmology, College of Medicine, the University of Arkansas for Medical Sciences, Fayetteville, AR USA; 6 Ophthalmic Center, the Second Affiliated Hospital of Guangzhou Medical University, Guangzhou, Guangdong China; 7 Department of Neurology, University of Miami Miller School of Medicine, Miami, FL USA

**Keywords:** Diabetic retinopathy, Homocysteine, Lutein, N-acetyl cysteine, Vitamins, L-methylfolate

## Abstract

Diabetic retinopathy (DR) is a form of microangiopathy. Reducing oxidative stress in the mitochondria and cell membranes decreases ischemic injury and end-organ damage to the retina. New approaches are needed, which reduce the risk and improve the outcomes of DR while complementing current therapeutic approaches. Homocysteine (Hcy) elevation and oxidative stress are potential therapeutic targets in DR.

Common genetic polymorphisms such as those of methylenetetrahydrofolate reductase (MTHFR), increase Hcy and DR risk and severity. Patients with DR have high incidences of deficiencies of crucial vitamins, minerals, and related compounds, which also lead to elevation of Hcy and oxidative stress. Addressing the effects of the MTHFR polymorphism and addressing comorbid deficiencies and insufficiencies reduce the impact and severity of the disease. This approach provides safe and simple strategies that support conventional care and improve outcomes.

Suboptimal vitamin co-factor availability also impairs the release of neurotrophic and neuroprotective growth factors. Collectively, this accounts for variability in presentation and response of DR to conventional therapy. Fortunately, there are straightforward recommendations for addressing these issues and supporting traditional treatment plans.

We have reviewed the literature for nutritional interventions that support conventional therapies to reduce disease risk and severity. Optimal combinations of vitamins B1, B2, B6, L-methylfolate, methylcobalamin (B12), C, D, natural vitamin E complex, lutein, zeaxanthin, alpha-lipoic acid, and n-acetylcysteine are identified for protecting the retina and choroid. Certain medical foods have been successfully used as therapy for retinopathy. Recommendations based on this review and our clinical experience are developed for clinicians to use to support conventional therapy for DR.

DR from both type 1 diabetes mellitus (T1DM) and type 2 diabetes mellitus (T2DM) have similar retinal findings and responses to nutritional therapies.

## Background

Food supplementation with vitamins, minerals, and nutraceuticals has been recommended by medical professionals for many decades [1]. It is a safe, simple, and inexpensive way to address risk factors and drivers of visual vascular disorders, including diabetic retinopathy (DR) [2–8]. DR is a form of microangiopathy. Elevated serum homocysteine (Hcy) increases microvascular damage [9, 10]. Reducing serum Hcy and oxidative stress of the mitochondria and cell membranes decreases ischemia and reduces end-organ damage to the visual system [11]. Though potential therapeutic targets are clear, the clinician is faced with a myriad of studies and single substance recommendations that are hard to grasp, explain to patients, or integrate with conventional diabetic and DR treatments.

This literature review summarizes the clinical benefits of nutritional supplements and medical foods for diabetes and DR, with emphasis on DR. We review the considerable literature supporting the vitamin and antioxidant interventions to reduce the risk and severity of vision loss. We review which forms of vitamins are optimal and the pitfalls of some synthetic vitamins. Finally, we distill these insights into simple, comprehensive recommendations for clinical practice. Time, usage, and future research will refine them, as clinicians gain experience with these new tools in their armamentarium to reduce the risk and severity of DR.

Current literature referencing supplementation was searched through PubMed using the search terms: vitamins, DR, hypertensive retinopathy, L-methylfolate, methylcobalamin, mitochondrial oxidative stress, MTHFR, and other closely related terms.

### Scientific Basis of Vitamin and Nutraceutical Therapy for Diabetes and DR and Vision

Vitamins and nutraceuticals have effects that directly increase the elasticity of blood vessels and metabolism [12–14]. Antioxidants are postulated to protect the body from free radicals and protect nitric oxide from inactivation [12, 13]. If the level of reactive oxidative species exceeds the capacity of antioxidant buffers, it creates oxidative stress. Measurements of oxidative stress can be an early indicator of hypertension, vascular disease, and diabetes [15].

Normally, nitric oxide promotes vascular health by controlling vascular tone (vasodilation), inhibiting platelet function, and preventing adhesion of leukocytes [16]. Reduced levels of nitric oxide result in endothelial dysfunction, causing inflammation, vasospasm, and thrombosis [17]. For example, vitamins C and E are antioxidants that limit oxidative stress by increasing nitric oxide. They also quench lipid peroxidation byproducts that injure cell membranes [18].

**Fig. 1:**
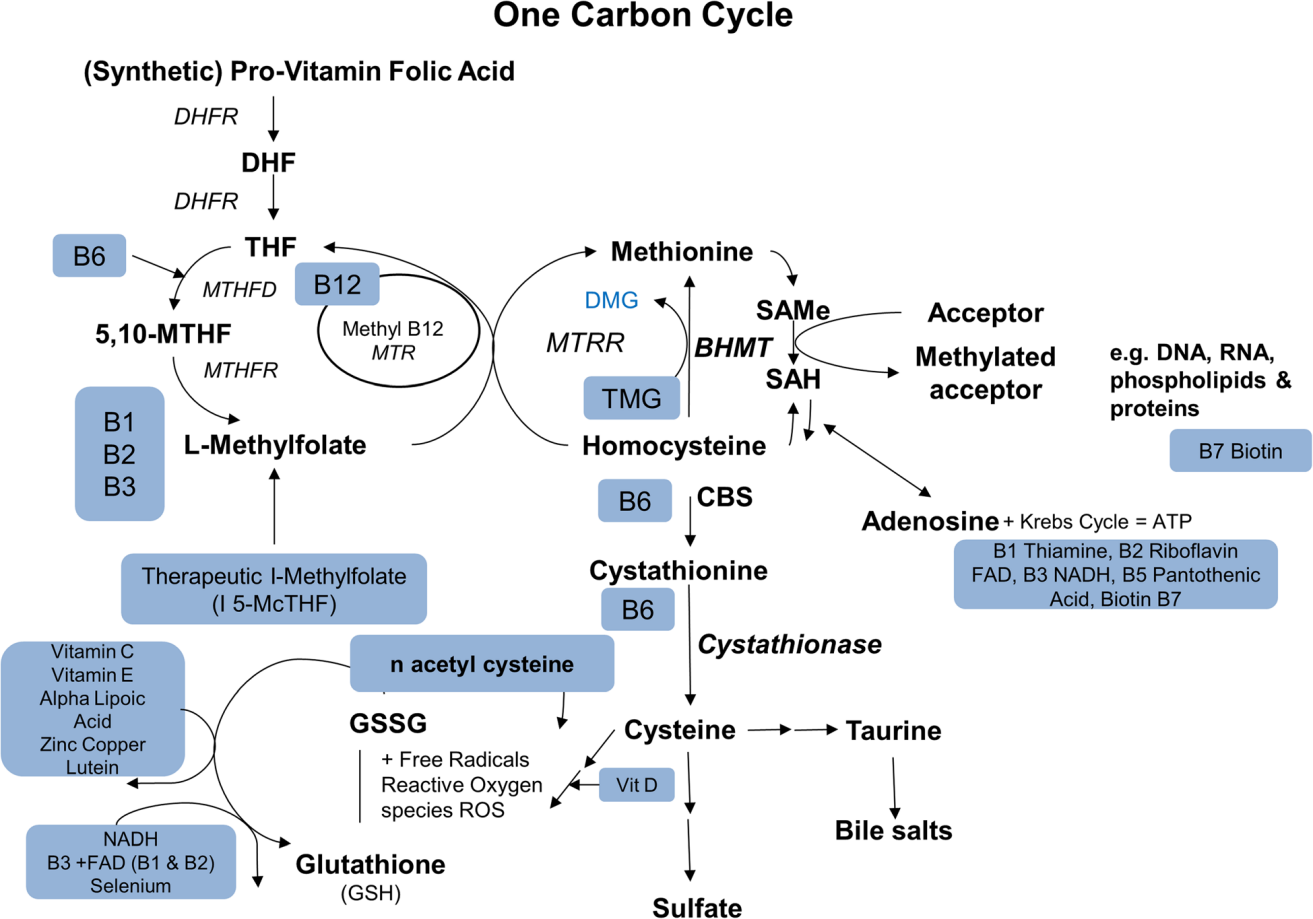
Vitamins and Cofactors of the One Carbon Cycle. DHF: Dihydrofolate; DHFR: Dihydrofolate reductase; THF: Tetrahydrofolate; MTHFD: Methylenetetrahydrofolate dehydrogenase; MTHFR: Methylenetetrahydrofolate reductase; MTR: 5-Methyltetrahydrofolate-Homocysteine Methyltransferase; MTRR: Methionine synthetase reductase; DMG: Dimethylglycine; TMG: Trimethylglycine; BHMT: Betaine-Homocysteine S-Methyltransferase; SAMe: S-adenosyl-L-methionine; SAH: S-adenosylhomocysteine; CBS: cystathionine β-synthase; GSSG: glutathione disulfide; NADH: nicotinamide adenine dinucleotide; FAD: flavin adenine dinucleotide; DNA: deoxyribonucleic acid; RNA: ribonucleic acid ; ATP: Adenosine triphosphate.

**Fig. 2:**
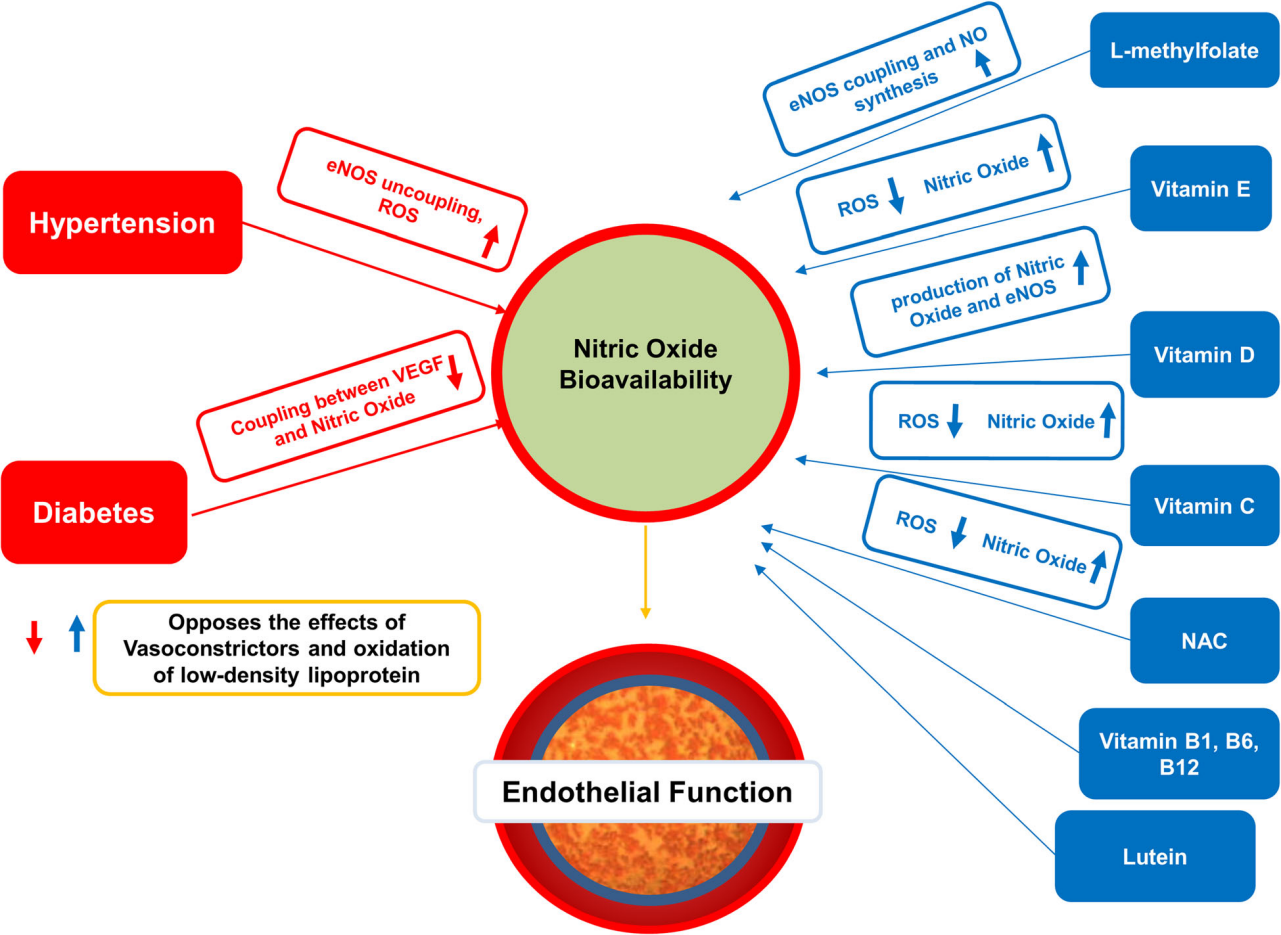
Effects of food supplements on vessel endothelial function in systemic vascular diseases. NOS: endothelial nitric oxide synthase; ROS: reactive oxygen species; VEGF: vascular endothelial growth factor; NAC: N-Aceytyl Cysteine.

DR is impacted by deficiencies and reduced function genetic polymorphisms of the B vitamin cofactors of the One Carbon Cycle (Fig. 1) and the Citric Acid Cycle (Fig. 2) [19–21]**.** Polymorphisms of the methylenetetrahydrofolate reductase (MTHFR) gene lead to reduced methylation, elevation of Hcy, reduction of nitric oxide, microvascular disease- particularly capillary endothelial injury and apoptosis, microaneurysms, leakage, ischemia, retinal atrophy, neovascularization, and vision loss [22].

Multiple studies have shown that vitamins C, D, E, B1, folate, B12, lipoic acid, lutein, n-acetyl cysteine, and betaine can improve endothelial function, protect neurons, lower blood pressure, and improve visual acuity [8, 23–35]. The retina is the recipient of these benefits.

## Main text

### Vitamins

#### Vitamin A and Carotenoids

**Vitamin A** is a group of animal-derived fat-soluble retinoids essential for cell growth, cell differentiation, immunity, and vision. In the eye, vitamin A (aka retinol), is a component of rhodopsin, the light-sensitive pigment. It is also necessary for healthy corneal and conjunctival membranes. Deficiency is common where there is generalized malnutrition and is associated with night blindness, conjunctival xerosis, and corneal ulceration, particularly with concurrent measles infection [36, 37].

Vitamin A deficiency decreases maintenance levels of nerve growth factor (NGF) and brain-derived neurotrophic factor (BDNF), which ordinarily protect the retina from oxidative stress injury and stimulate repair. Repletion of vitamin A restores NGF and BDNF levels in the brain [38].

**Lutein and Zeaxanthin** are water-soluble plant-based carotenoids that easily cross the blood-brain and blood-retina barriers [39]. They are essential for vision but cannot be synthesized in the human body. Concentrated in the macula lutea, they act as powerful antioxidants stabilizing cell membranes and protecting from oxidative stress. They are believed to protect against age-related macular degeneration (AMD) and DR [40].

The Age-Related Eye Disease Studies (AREDS), are an important series of studies conducted by the National Eye Institute investigating how multi-vitamin antioxidant complexes affect AMD and other eye diseases. The first study, AREDS report 8, looked at the progression of moderate AMD [5].

Seddon et al. found that AMD protection was linear with lutein intake and that 6 mg/day or more of lutein was required [41]. Subsequently, the AREDS 2 study replaced beta carotene of the AREDS formula with 10 mg of lutein and 2 mg of zeaxanthin. AREDS2 found that patients with the lowest baseline intakes benefited the most [42].

These studies proved that optimal combinations of crucial nutrients, including lutein and zeaxanthin, could slow the progression of an inexorable degenerative disease like AMD. These findings were initially controversial but are cost-effective and have stood the test of time [43–45]. Evidence suggests that lutein supplementation also increases BDNF, preventing neurodegeneration, and preserving the electroretinograms [28, 46, 47].

Several studies extended the AREDS concepts to DR [48–50]. Brazionis et al. reported that similar to AMD, higher lutein, and zeaxanthin levels were associated with significantly lower odds of DR [51]. A randomized trial of several antioxidants showed lutein could delay the progression of DR over five years [48]. An interventional study reported that intake of 10 mg/day of lutein improved contrast sensitivity, glare, and visual acuity in patients with non-proliferative DR [52]. A two year trial of 10 mg lutein + 12 mg zeaxanthin in diabetic patients without DR showed improved retinal response density on multifocal electroretinography and a mild non-edematous increase in foveal thickness [53]

These two carotenoids show benefit for AMD and DR [29]. The optimal dosage of them may be higher than 10 mg of lutein and 2 mg of zeaxanthin. Many patients currently self-administer high doses of lutein and zeaxanthin, with no apparent harm. Toxicology studies are reassuring [54]. **L**ong-term studies with larger sample sizes will help clarify optimal dosing.

#### B Vitamins: B1, B2, B3, B5, B6, Folate, B12

B vitamins are a group of essential water-soluble co-factors that regulate key cellular metabolic processes. These active metabolic pathways are critical to all cells (Figs. 1 and 2) [55].

Genetic polymorphisms and B vitamin insufficiencies wreak havoc on the homeostatic mechanisms of intermediate metabolism, particularly the One Carbon Cycle, which maintains healthy vascular endothelium by converting Hcy to methionine. Other functions include mitochondrial free radical quenching, blood pressure regulation (nitric oxide- norepinephrine), white matter tract signal conduction (synthesis of myelin), sleep (serotonin-melatonin diurnal cycles), attention and memory (dopamine and norepinephrine), and mood (serotonin). These are critical functions for neural, vascular, and visual function. They are at the root of many chronic illnesses as causative or aggravating conditions, which negatively impact health [19, 56–59]. A deficiency of any one B vitamin decreases the efficiency of all linked processes throughout the One Carbon pathway [19].

B-vitamin deficiencies, insufficiencies, and reduced function genetic polymorphisms first show their effects in the mitochondria of tissues with the highest metabolic activity [55].

The retina has the highest metabolic activity and metabolic stress in the human body. The inner retina has the highest metabolic vulnerability to ischemia [60–64]. The following is a review of the impact of individual B-vitamins on metabolic issues of DR.

##### Vitamin B1 (Thiamin)

Thiamin is a potent free radical scavenger that regulates intracellular glucose and prevents polyol pathway activation, which is induced by high intracellular glucose [65, 66]. Hyperglycemic-induced dysfunction of the polyol pathway is thought to induce DR in rats and humans [67, 68].

High serum levels of thiamin protect the vascular endothelium from advanced glycation end products injury [30, 69, 70].

Thiamin supplementation at high doses of 50-100 mg/day is safe and useful for neuroprotection as well as the treatment and prevention of end-organ vascular damage, including DR and diabetic nephropathy. Toxicity is so low that no Upper Limits (UL) have been proposed. Thiamin supplementation offers a very useful low-cost low-risk intervention for treating DR (Fig. 2) [71–73].

##### Vitamin B2 (Riboflavin)

Riboflavin is a flavonoid vitamin essential for intermediate metabolism, energy production, and mitochondrial function [74]. Riboflavin, as flavin adenine dinucleotide, is an essential cofactor for synthesizing L-methylfolate, the methyl source for methylcobalamin, which lowers Hcy [58].

The common C677T polymorphism of the MTHFR enzyme has impaired sensitivity to flavin adenine dinucleotide, reducing L-methylfolate synthesis. This causes elevated Hcy resulting in hypertension and vascular disease [40]. Increased serum Hcy in humans has been associated with loss of retinal thickness measured by optical coherence tomography and an increased incidence of DR (Fig. 1) [75].

Human supplementation with riboflavin increases L-methylfolate synthesis, lowers Hcy, and lowers blood pressure [58]. In a murine model, riboflavin supplementation increases glucose uptake and ameliorates oxidative stress. Riboflavin supplementation thus appears to protect the retina from oxidative stress, hyperglycemia, and Hcy-induced injury [59, 76].

Riboflavin supplementation also increases BDNF expression in a murine model [77]. Low levels of BDNF are associated with impaired glucose metabolism. BDNF is higher in prediabetic patients than in diabetic patients, suggesting neuroprotective benefits for patients with insulin resistance and pre-diabetes [78].

Optimal riboflavin dosages are not established for DR, but long-term treatment with 400 mg/day is common for migraine, and toxicity is so minimal that there is no UL established [39].

##### Vitamin B3 (Niacin)

Niacin is an essential water-soluble B-vitamin. High dose supplementation of niacin may cause or aggravate diabetes. Pharmacologic doses are used for lipid control 1000-3000 mg/day, with questionable benefit and an increased risk of impaired glucose tolerance and insulin resistance, hepatic toxicity, and all-cause mortality (Fig. 2) [79, 80].

Cystoid macular edema risk is increased with high dose niacin supplementation [81]. On the positive side, in a diabetic animal model, niacin supplementation increased endothelial growth factors, promoted migration, sprouting, and survival of endothelial cells and mediated vascular remodeling following occlusion of the middle cerebral artery [82].

Retinal vein occlusion (RVO) prevalence is increased in patients with diabetes and may present with DR [83]. Three small studies suggest that niacin supplementation hastens the resolution by vasodilation, and visual acuity may decrease when niacin is withdrawn [84].

The above studies suggest that niacin intake should remain between the recommended dietary allowances (RDA) and tolerable upper intake level (UL) (14-30 mg/day) unless the patient is monitored for an increased risk of diabetes [79]. There may be a benefit for high niacin for cholesterol where statins fail, in the acute post-stroke recovery phase, or for the treatment of recalcitrant cystoid macular edema, but the macula, blood sugar level, and liver enzymes must be monitored carefully. Except in these special conditions, we do not recommend long-term niacin supplementation above the UL.

##### Vitamin B5 (Pantothenic Acid)

Pantothenic acid is important for fatty acid metabolism, particularly in the Citric Acid Cycle. It is abundant naturally in foods and seldom requires supplementation (Fig. 2) [85]. In an animal model, 300 mg/kg supplementation of dexpanthenol restored endothelial function, improved antioxidant status, and decreased blood glucose level without side effects [86].

##### Vitamin B6 (Pyridoxal 5’ Phosphate, PLP, P5P)

Vitamin B6 is a cofactor for many key metabolic activities, including One Carbon Cycle methylation and Hcy metabolism (Fig. 2) [19, 21, 87]. The NHANES studies of B6 in the US population suggest that inadequate serum B6 levels occur in 10-40% of surveyed groups despite widespread use of multivitamins [88]. When MTHFR polymorphisms are present, deficiencies in B6 increase Hcy. Elevated Hcy is a risk factor for DR [10, 89, 90].

Vitamin B6 deficiency contributes to pancreatic islet cell autoimmunity resulting in type I diabetes [91, 92]. A large cohort of Japanese type 2 diabetics was followed for eight years monitoring vitamin B6 intake and the development of retinopathy. Lower intake, particularly in the lowest quartile, was associated with an increased incidence of DR [93].

There are several forms of B6. The natural active form is P5P. It appears to be safe in pharmacological doses. However, other forms of B6, such as the commonly encountered pyridoxine if given in pharmacological doses, may cause a peripheral neuropathy indistinguishable from B6 deficiency. This is caused by competitive inhibition of the enzyme that converts it into P5P. Thus, P5P is the form that is the safest and most efficient for Hcy intervention [94].

Supplementation with B6 optimally as P5P may reduce the risk of developing diabetes, and DR. Studies should be designed to verify this and optimize dosing because such intervention would be high yield, low risk, and affordable.

##### Vitamin B7 (Biotin)

Glucose metabolism and insulin resistance in T2DM cause dysregulation of glucose-6-phosphatase, hepatic enzymes phosphoenolpyruvate carboxykinase (PCK1, PCK2), and glucose kinase (GCK) metabolism resulting in excess glucose release into the blood. GCK activity is particularly reduced in T2DM, negatively correlating with HbA1c [95]. Animal models show that biotin regulates GCK and that supplemental biotin improves postprandial glucose response by affecting GCK and PCK1 [96, 97]. Biotin should be stopped 72 hours before testing for high-sensitivity troponin T, thyroid-stimulating hormone, follicle-stimulating hormone results, triiodothyronine, or vitamin D [98]. Biotin is safe and inexpensive, without an assigned UL (Fig. 2) [99].

##### Vitamin B9 (Folate)

Folates are essential water-soluble compounds that serve vital biochemical pathways in every cell of the body. L-methylfolate is the natural substrate for single-carbon methyl transfers in the synthesis of amino acids and nucleic acids (DNA, RNA). It indirectly regulates neurotransmitter and nitric oxide synthesis. L-methylfolate with methylcobalamin converts Hcy to methionine necessary for the synthesis of S-adenosyl-methionine, a key methyl donor for the synthesis and regulation of DNA required for protein synthesis and cell division (Fig. 2) [100].

At the cellular level, elevated Hcy disrupts the retinal blood barrier and increases pigment epithelial cell inflammatory cytokines, which cause retinal apoptosis [101]. Even mild hyperhomocysteinemia is a risk factor for insulin resistance in healthy subjects. The results of the Framingham Offspring Study and other similar studies suggest that it is a cause and marker for pre-diabetes [102–104].

The MTHFR enzyme is essential for adding the methyl group to upstream folates. Reduced activity MTHFR gene polymorphisms are common and impaired in their ability to generate L-methylfolate [105]. They are associated with elevated blood pressure and Hcy, as well as increased incidence and progression of DR [58, 106, 107].

L-methylfolate supplementation effectively restores impaired endothelial-dependent vasodilation and enhances endothelial health by converting Hcy to methionine, regardless of dietary deficiencies or genetic polymorphisms that inhibit folic acid processing or L-methylfolate synthesis [31, 104, 108–111].

L-methylfolate is the reduced natural bioactive form of folate. Folic acid is active only to the degree it has been converted to L-methylfolate [67]. The Institute of Medicine (IOM) reports no toxicity for the natural folates and has not issued tolerable ULs for natural folates. However, the IOM has set the UL for folic acid at 1.0 mg/day for adults [100, 108]. Reynolds has raised concerns that folic acid may cause neurologic injury when there is B12 deficiency [112]. Selhub and Rosenberg reviewed the evidence that a high intake of folic acid is linked to impaired cognition, memory, executive decision making, and retinoblastoma [113]. Folic acid poisoning can be fatal [114]. Any dosing above 1.0 m/day should be with L-methylfolate.

Elevated Hcy increases the risk of hypertension, hypertensive retinopathy, diabetes, and DR [115, 116]. L-methylfolate can be co-administered with vitamin B12 as methylcobalamin efficiently lowers Hcy [117]. Lowering Hcy with folate increases blood flow and perfusion [118]. The use of L-methylfolate with B2, B6 as P5P, and B12 to lower Hcy provides a safe, simple, and inexpensive to reduce and reverse DR and other diabetic end-organ diseases.

#### Vitamin B12 (Cobalamin)

Vitamin B12, cobalamin, is a complex water-soluble cofactor serving critical functions for cell synthesis, DNA regulation, Hcy metabolism, myelin synthesis, nerve growth, and neuron maintenance; all of which impact vision and DR. Methylcobalamin, one of two active forms, readily donates a methyl group to lower Hcy converting it to methionine [9, 57, 119–123]. Methylcobalamin, through methionine synthesis, regulates the synthesis of DNA, key amino acids, and proteins [124]. Elevated Hcy is associated with reduced cerebral blood flow, reduced retinal blood flow, and reduced caliber of the central retinal artery, vascular endothelial growth factor (VEGF) expression, and DR [125–129].

High dose methylcobalamin is efficient for reducing Hcy because it possesses a ready-to-donate methyl group and would have a beneficial effect on those markers (Fig. 1) [130].

Diabetes leads to small vessel disease of the brain and retina, with ischemia contributing to the pathogenesis of DR [131]. Initial mitochondrial dysfunction and Müller cell impairment are followed by structural loss of capillary endothelium, neurons, and photoreceptors. This process is visible in the eye as microaneurysms, exudates, cotton wool spots, capillary drop out, retinal edema, and retinal atrophy [132, 133].

Animal models suggest that treatment with NGF for retinal inflammation and neovascularization could be the next major therapeutic advance in tandem with anti-VEGF therapy. Supplemental B12 increases the release of NGF and BDNF [134]. Combined therapy increases retinal cell survival, rhodopsin expression, and neurite outgrowth in photoreceptors [135]. This may safely and inexpensively be accomplished by maintaining B12 levels in the high normal range. Raising neurotrophins by treating with B12 offers another opportunity for clinicians to reduce long-term vascular complications of DR.

B12 insufficiency and deficiency is common and has many causes [136]. Active transport B12 absorption from food requires intrinsic factor, secreted by the gastric parietal cells, in an acid milieu with intact small intestinal villi [137]. Metformin, Glipizide, and omeprazole, common medications for diabetic treatment impair B12 uptake. Passive transport uptake for B12 is about 1%; thus, high doses of active B12 bypass active transport mechanisms and are less prone to iatrogenic or disease state malabsorption [138–140]. Eussen et al. studied oral dosing of B12, concluding that satisfactory passive transport was dose-related and required at least 200 times greater than the RDA of 2-4 μg/day. Oral doses of 500-1000 μg/day, at least, were recommended [141].

B12 therapy, through enhanced reduction of Hcy and nerve growth factor release, is a neglected, inexpensive opportunity for clinicians to reduce vascular ischemia of the retina and to reduce the risk and severity of DR [9, 57, 120–122].

Recently, concerns have risen about the toxicity of cyanocobalamin, especially in people with diabetes. Methylcobalamin in high doses is non-toxic, even when advanced diabetic nephropathy is present. Methylcobalamin appears to be safer and more effective than cyanocobalamin in reducing Hcy, even with advanced renal disease [130, 142].

#### B-Vitamin Summary: Diabetes, DR, Hcy, Blood Flow Therapies

Monotherapy is the ordinary trend in medicine. However, in strategically formulated nutritional therapy, B-vitamin combinations at high dosages seem to have better outcomes than vitamin monotherapy. Martin et al. linked Hcy elevation to occlusive retinal vascular disease and proposed therapeutic intervention with vitamins B6, B9 (folate), and B12 [143]. Vitamins B2, B6, B9 (folate), and B12 are the primary cofactors for One Carbon Metabolism and are important for Hcy methylation and regulation [144]. Multivitamin complexes containing B1, B2, B6, L-methylfolate, and B12 have shown benefit for DR in human and animal trials [145–147].

#### Vitamin C (Ascorbic Acid)

Vitamin C is water-soluble and essential for regenerating other antioxidants such as vitamin E and glutathione [148, 149]. Vitamin C administration lowers blood pressure in patients with essential hypertension [150]. Human and diabetic animal trials have found that oral vitamin C reduces capillary endothelial dysfunction [32, 151]. Patients with proliferative DR have a 10-fold lower vitreous ascorbate concentration and an increased tendency to diabetic macular edema [152]. Vitamin C taken with statins reduces non-proliferative DR, in a dose-dependent fashion more than statins alone [153].

#### Vitamin D

Vitamin D is a group of fat-soluble vitamin secosteroids essential for calcium absorption, deposition, and regulation, which in turn regulate many important processes. The prevalence of vitamin D insufficiency in the US is above 40%, higher in the elderly, and in some ethnic groups [154]. Most supplements are vitamin D2 or D3, which are storage forms collectively referred to as "vitamin D." Serum testing for vitamin D 25 (OH) D2 + D3 measures body stores [155]. 1,25 (OH) D is the active vitamin synthesized by the kidneys as needed. It is seldom supplemented except in renal failure states. Vitamin D and calcium also regulate tear film mucin release and stability [156, 157].

Intestinal folate absorption and transport across the blood-brain barrier are upregulated by vitamin D [158, 159]. Since folates require sufficient available vitamin D to be effective, and vitamin D deficiency is so widespread, if folate is to be given therapeutically, it should be given with enough vitamin D to ensure optimal absorption and CNS transportation. Given widespread vitamin D deficiency, we suggest co-administration with the UL of 4000 IU vitamin D_3_ daily with any folate therapy unless serum vitamin D is known to be above the 50^th^ percentile.

Vitamin D sufficiency is essential for insulin release, insulin sensitivity, reduction of inflammation, and reduction of arterial stiffness [155, 160–165]. Recently, optimal vitamin D levels have been shown to be important to reduce the risk and severity of DR [166]. Vitamin D plays a role in pancreatic β-cell function [167]. Deficiency reduces insulin sensitivity and increases the risk of atherosclerosis, CVD, T2DM, and hypertension [168–170]. 1,25–dihydroxy vitamin D triggers the secretion of insulin by stimulating pancreatic beta cells [164, 165]. Clinical trials had shown significant improvements in insulin sensitivity and HbA1c when patients were given vitamin D_3_ [171, 172].

Mutlu et al. reported that lower vitamin D was associated with retinal microvascular damage after studying the associations in 5675 participants with diabetes [173]. Vitamin D deficiency is linked to T1DM [174]. Vitamin D deficiency is also linked to T2DM and supplementation, which has been shown to decrease C-reactive protein, hs-CRP [175].

Serum levels of 25-hydroxy vitamin D above 30 ng/ml reduce the odds of DR [166, 176]. Vitamin D supplementation reduces intracellular reactive oxygen species decreasing VEGF expression [7].

Low serum vitamin D levels among patients with DM are associated with a higher risk and severity of DR for all the above reasons. Cumulatively, this suggests that vitamin D supplementation is beneficial to reduce the risk and severity of DR,

#### Vitamin E

Vitamin E is an amber lipid-soluble antioxidant associated with low-density lipoprotein. It primarily functions as a peroxyl radical scavenger involved in long-chain fatty acid stabilization of cell membranes [177, 178]. Vitamin E supplementation quenches free radicals and reduces retinal oxidative stress in the retina [178, 179].

Vitamin E supplementation reduces moderate blood pressure abnormalities, particularly systolic pressure [180–182]. Elevated blood pressure is a risk factor for both the incidence and severity of DR [183]. A careful randomized, double-masked, placebo-controlled crossover trial at the Joslin Institute established that for patients with T1DM of fewer than ten years duration, vitamin E supplementation of 1800 IU daily improved retinal blood flow [184]. Oxidative stress, which is elevated in DR, is decreased after treatment with vitamin E [185]. Vitamin E, when administered alone, has modest benefits on blood pressure and blood flow, which are salutary for diabetes and DR patients [186]. It appears vitamin E has a greater benefit when co-administered with vitamin C [187]. Studies suggest that natural vitamin E complex, not the racemic synthetic *dl* mixtures, is more potent and more likely to be beneficial [188].

#### Zinc

Zinc is an essential co-factor for cell division, DNA synthesis, immune function, as well as the metabolism of carbohydrates and proteins. Moderate zinc deficiency is common [189–191]. Zinc deficiency is associated with the progression of chronic disease states such as metabolic syndrome, diabetes, diabetic microvascular complications, and DR [192–194].

The Nurses' Health Study found a 20% difference in risk of diabetes between the highest and lowest zinc intake quintiles [195]. Low serum zinc levels are correlated with the duration of diabetes, elevated HbA1c, hypertension, and microvascular complications. Serum zinc levels fall progressively with increased duration of diabetes and severity of DR [194].

A trial of zinc supplementation in a murine T2DM analog improved glucose intolerance, insulin resistance, obesity, and hypertension [196]. This may be in part due to zinc protection against lipid peroxidation and pericyte protection. Increasing zinc also reduced ischemic inflammation while decreasing VEGF [192].

A rat model of DR was studied using an AREDS-based micronutrient supplementation, including zinc. Capillary deterioration over time was prevented with the AREDS formula despite similar hyperglycemia between treated and control groups [50]. A human trial should be done to establish optimum zinc levels for the treatment of DR.

#### Lipoic Acid (LA, Alpha Lipoic Acid, ALA, Thioctic Acid)

Lipoic acid also referred to as alpha-lipoic acid, is an important cofactor for mitochondrial metabolism. LA is needed to generate acetyl Co-A, the fuel of the Krebs Cycle, and the core of energy-generating metabolism [197, 198]. LA scavenges reactive oxygen species, enhancing the effects of such endogenous antioxidants as glutathione, vitamin C, and E by recycling. Lipoic acid supplementation increases available glutathione [199].

LA administration protects the retina, particularly the ganglion cells and pigment epithelial cells from ischemia and apoptosis [200, 201]. LA decreases hyperglycemia and hyperglycemic vascular endothelial dysfunction in T2DM patients [202, 203]. It reduces VEGF expression and is protective of the retinal ganglion cells and capillaries in animal models of DR [34, 204]. A small controlled study showed increased contrast sensitivity in patients with T1DM and T2DM who were supplemented with oral LA [205].

Supplementation is beneficial. It shows benefits for ischemia and oxidative stress. LA appears to be safe and well-tolerated at 600 mg/day as a dietary supplement. When used for diabetic polyneuropathy, it is effective and has fewer side effects than commonly prescribed medications [206]. LA represents a new opportunity for patients with diabetes, and DR. More research is strongly encouraged.

#### N-Acetyl Cysteine (NAC)

N-acetyl cysteine is a thiol antioxidant precursor to glutathione [207]. Glutathione is neuroprotective and retina protective [208, 209]. Cysteine availability is often the rate limiter for glutathione synthesis, and thus supplementing with NAC increases net glutathione levels, which is useful in disease states characterized by ischemia, increased oxidative stress, and reduced available glutathione [210, 211].

Hyperhomocysteinemia has been shown to increase reactive oxygen species in the retina, altering the blood-retinal barrier of human retinal endothelial cells [212]. Chronically elevated Hcy eventually causes vaso-occlusive retinopathy with retinal atrophy [75, 213].

Long term administration of NAC reduces reactive oxygen species in the retinal mitochondria, and decreases VEGF expression and proliferative retinopathy in diabetic animal models [214]. NAC restores the tight junctions reducing vascular leakage [212]. Ischemia-induced retinal pigment epithelial cell, ganglion cell, and photoreceptor apoptosis are reduced when NAC is present [35, 209, 215, 216]. It also reduces the expression of VEGF and Icariin-1, free radical species, ischemia, and structural changes of retinopathy [214]. Animal models suggest that it may decrease retinal detachment in proliferative vitreoretinopathy [217].

Therefore, NAC is beneficial at the cellular level to prevent retinal neuron and photoreceptor apoptosis and death while preserving the visual function and structure of the retina and optic nerve.

#### Trimethylglycine (TMG, Betaine)

TMG is a trimethylated dietary amino acid derivative primarily obtained from sugar beets. Betaine is not a vitamin because it can be synthesized in small amounts by methylating choline in the mitochondria in young adults. Though betaine has no RDA established, choline has an RDA. Diets are frequently low in both [218–221].

Homocysteine is at the crossroads of the One Carbon Cycle [221, 222]. The betaine-homocysteine methyltransferase pathway is an alternative pathway for converting homocysteine into methionine via the methylfolate-B12-homocysteine methylation pathway. They work together to keep serum Hcy low and methionine at an optimal level [223]. The main sources of the One Carbon Cycle methyl groups are choline, L-methylfolate, methylcobalamin, dimethylglycine, and TMG. Higher intake of betaine decreases inflammatory markers and serum Hcy [221, 224, 225]. Betaine naturally concentrates in the brain, liver, and kidneys, where it prevents the accumulation of Hcy and decreasing inflammation (Fig. 2) [218].

Betaine is used as a secondary treatment for elevated Hcy in patients who do not respond sufficiently to B6, folate, and B12 supplementation [226]. The human average dietary intake of betaine is 500-2000 mg/day. In a study of betaine reduction of Hcy, low dose additional supplementation with betaine anhydrous of 500-3000 mg/day lowered Hcy levels in healthy subjects, with a small further drop at 6000 mg/day [227].

Homocysteine elevation is associated with increased inflammation and decreased blood flow in the brain and retina, causing small vessel disease of the central nervous system (CNS), increased VEGF secretion, endothelial dysfunction, proliferative and non-proliferative retinopathy, as well as diabetic macular edema [126, 129, 228–232]. Betaine, along with vitamins B2, B6, folate, and B12, is very effective in reducing Hcy levels and reversing the triggers of hyperhomocysteinemia, a driver for DR [58, 233, 234].

### Multivitamins and Nutraceuticals: Monotherapy or Polytherapy?

Drug-based therapies tend to be monotherapies with a single drug for a single target. Nutritional deficiencies are often multi-faceted. Genetic polymorphisms and nutritional deficiencies tend to have a broad impact on complex systems, such as the One Carbon Methylation Cycle or the Citric Acid Cycle, where there are many cofactors and substrates. Targeting more than one place in these cycles seems efficient for correcting cycle and functional imbalances (Figs. 1 and 2).

Multivitamins mineral complexes and B-complex multivitamins have long been used to treat and prevent multiple nutritional deficiencies because mono-deficiencies are rare [235–237]. In the central nervous system, where metabolic rates are high, there is a synergistic benefit to using several of the B-vitamins together [21].

For people with diabetes, B vitamin serum concentrations are lower, possibly due to higher renal clearance and lower reabsorption [238]**.** This leads to further deficiencies and the need for even higher B-vitamin levels.

Active transport uptake of vitamins and minerals have limits that may lead to a deficiency or prevent optimization [239]. However, with high doses, absorption and transport are passive, and dose-related [141, 240, 241]. Thus, there is precedence for this high dose multivitamin approach applied to conditions of the retina and central nervous system [19, 242]. Fortunately, long term experience has shown a few complications with this approach because of their low toxicity [243, 244].

The AREDS/AREDS2 studies showed powerful benefits and excellent safety for macular degeneration using a high dose combination of specific vitamins, minerals, and lutein plus zeaxanthin. No human trials have been done with the AREDS formulation for DR, however, Kowluru et al. studied diabetic rats given a diet that included the AREDS micronutrients. They found that the AREDS micronutrients decreased the accumulation of acellular retinal capillaries. Evidence of diabetic oxidative damage and retinal levels of manganese superoxide dismutase were decreased. Diabetes-induced increased nitric oxide synthetase was blocked. This mechanistically supports the use of such vitamin antioxidant combinations for DR [44, 50].

Folate and vitamin B12 intake and absorption decline with age and diabetes [238, 245, 246]**.** As discussed previously, they are essential to control homocysteine. There is also evidence that both natural aging and AMD are associated with homocysteine elevation and low folate and B12 serum levels [247]. Choriocapillaris endothelial loss slowly proceeds with age, increasing with AMD. This loss long predates visual detection [248, 249].

If the choriocapillaris endothelium in AMD is injured by hyperhomocysteinemia the same way as are the retinal capillaries in DR, this would suggest a unifying approach. There is such evidence. The Women's Antioxidant and Folic Acid Cardiovascular Study (WAFACS) trial was a large randomized, double-blind, and placebo-controlled study that examined the benefits of high dose B6, folate, and B12 in women at high risk of cardiovascular disease. With an average follow-up time of 7.3 years, after two years, a reduction of AMD emerged, reaching 40% by the end of the trial. This exceeds the benefit of the AREDS2 trial. The investigators proposed homocysteine reduction to account for this [250]. Adding high doses of B-vitamins to an AREDS2 type formula might further benefit macular degeneration.

The use of higher dose B complex multivitamins has also been shown to reduce the risk of all strokes and improve brain health in the Hope 2 trial [19, 251].

### Medical Foods

Medical foods are a category of nutritional interventions to which ophthalmologists have had little exposure. They occupy a place between foods, dietary supplements, and prescription drugs. All ingredients of medical foods must be generally recognized as safe, Generally Recognized as Safe (GRAS), meaning that the FDA has approved them as safe for human consumption. FDA oversight for claims, purity, and manufacturing is more rigorous than dietary supplements. Labeling must be accurate, unlike the reality of dietary supplements [252–256].

The FDA restricts the use of medical foods to patients under the direct supervision of a physician. They are for situations in which diet alone is insufficient to obtain what is needed. Particularly, the FDA intends them to address inborn errors of metabolism caused by reduced function genetic polymorphisms resulting in or contributing to disease states. An example pertinent to DR is the elevation of blood pressure and serum Hcy due to MTHFR gene polymorphisms that impair the One Carbon Cycle [257].

Medical foods addressing Hcy elevation due to inborn errors of metabolism, do so by providing high doses of active forms of vitamins B6, L-methylfolate, methylcobalamin, or trimethylglycine. They work by donating a methyl group to homocysteine, thus converting it to methionine, reducing mitochondrial oxidative stress, and protecting the vascular endothelium. Over time, this reduces end-organ damage, such as retinopathy. Three medical foods are useful for treating elevated Hcy, reduced blood flow, and retinal ischemia.

**Metanx®** was developed to support neurovascular regeneration for diabetic peripheral neuropathy associated endothelial dysfunction in patients with inherited disorders of Hcy metabolism with elevated Hcy. Metanx® consists of 3.0 mg of L-methylfolate, 35 mg of pyridoxal 5’- phosphate P5P, and 2 mg of methylcobalamin [258]. Metanx® utilizes P5P, which is the non-toxic active form of vitamin B6 [94]. L-methylfolate is the natural, non-toxic active form of folate and methylcobalamin is the natural, non-toxic methylated active form of cobalamin. These are the most effective, least toxic vitamin forms for reducing Hcy, increasing blood flow, and raising brain-derived neurotropic factors for neuroprotection [117, 259, 260].

**Cystadane®** was developed for the long term management of elevated Hcy. Cystadane® is pure anhydrous betaine powder for the preparation of an oral solution [261]. Betaine (TMG), is another methyl source. Cystadane lowers homocysteine through the betaine-homocysteine methyltransferase pathway [223]. It is useful when B-vitamin therapy alone is insufficient to bring Hcy levels below 9 μmol/L.

**Ocufolin®** was developed to reduce retinal ischemia and retinopathy in patients with the common MTHFR polymorphisms. The ingredients collectively address critical metabolic pathways with vitamins and antioxidant co-factors, which lead to Hcy elevation, ischemia, and oxidative stress in those patients [262].

### Studies of Medical foods for DR

Liu et al. found that Metanx^TM^ inhibited ocular oxidative stress, inflammation, and protected against diabetic spatial frequency defects in a ten-month study of mice with DR [145]. In a six month observational study of human subjects, Smolek et al. found that Metanx^TM^ reduced non-proliferative DR (NPDR), particularly foveal edema, retinal thickness, and improved the mean threshold retinal sensitivity [146]. The protective mechanism is most likely due to decreasing Hcy, which appears to constrict the central retinal artery [126, 232].

Schmidl et al. found that Ocufolin® reduced Hcy levels 30% in a trial of type 1 and 2 diabetics [263]. Wang et al. published a case series of eight patients with DR treated with Ocufolin^TM^ or a similar formulation (Eyefolate^TM^). They showed visible improvement in retinopathy even in longstanding cases [262].

Based on published studies, it is possible to come to some recommendations for reducing the risk and severity of DR. It is helpful that the recommendations for DR mirror those for diabetes and macular degeneration generally, and for reducing Hcy, hypertension, and increasing BDNF and other nerve growth neurotropic factors so that many benefits flow from similar treatments.

## Conclusions

We have identified vitamin deficiencies, antioxidant deficiencies, and the reduced function gene polymorphisms of MTHFR as common risk factors for hyperhomocysteinemia, neurotrophic factor depletion, and DR. We also note that their physiological mechanisms overlap and that the treatments are similar. Most chronic diseases are worsened by a deficiency of any essential nutrient. As we have seen, age, diet, and many factors further impair the absorption and utilization of these nutrients. This paper has identified several vitamins, minerals, and nutraceuticals, which are useful to address this situation. They include lutein, zeaxanthin, vitamin C, vitamin D, vitamin E, zinc, copper, alpha-lipoic acid, n-acetylcysteine, and complexes of B1, B2, B6, L-methylfolate, and methylB12. Some of these were also shown to be beneficial for AMD in the AREDS/AREDS2 trials. Addressing Hcy, raising BDNF and other neurotrophic factors, reducing oxidative stress and inflammation, while increasing blood flow is a low hanging fruit for the clinician who wishes to lower the burden and alter the course of disease in patients with DR.

It is possible for a patient and clinician to assemble all these individually. However, cost and logistics may be concerns. The AREDS2 formulation of the National Eye Institute was a major improvement, bringing together copper, zinc, lutein, zeaxanthin, vitamin C, and vitamin E for macular degeneration in a convenient, affordable, and effective format. AREDS2, however, does not address blood flow, ischemia, or Hcy reduction. It does not maximally increase glutathione, nor is it intended for retinal vascular diseases such as DR.

Similarly, medical foods that address common inborn errors of metabolism, homocysteine elevation, reduced blood flow, and ischemia makes it simpler for the patient and clinician to obtain these benefits. The FDA holds medical foods to higher standards of manufacturing, labeling, and safety than dietary supplements, resulting in precise, consistent dosing which physicians need for addressing serious health conditions.

These opportunities support without conflicting with conventional therapy for DR. While further studies are needed to determine optimal formulations and appropriate usage, clinicians should feel comfortable about the safety and utility of these modalities.

## Data Availability

Not applicable.
